# Embedding research codesign knowledge and practice: Learnings from researchers in a new research institute in Australia

**DOI:** 10.1186/s40900-022-00392-4

**Published:** 2022-12-07

**Authors:** Sara Javanparast, Sally Robinson, Alison Kitson, Joanne Arciuli

**Affiliations:** 1 Caring Futures Institute, College of Nursing and Health Sciences, Flinders University, Adelaide, Australia; 2 Present Address: Research Centre for Palliative Care, Death and Dying, College of Nursing and Health Sciences, Flinders University, Adelaide, Australia

**Keywords:** Research codesign, Research institute, University, Research end-user, Capacity building, Research impact.

## Abstract

**Background:**

Research codesign is generally defined as end-users’ involvement in planning, implementation, and evaluation of projects. Recently, there has been a growing interest in codesign to maximise research acceptability, applicability, and impact and to address longstanding issues around power and depth of involvement. Frameworks have been developed to assist in understanding research codesign processes at a project level. However, little is known about how university based researchers construct or adopt a coherent approach to sustain research codesign in governance, methodological approaches, and practice. This study investigated the perspectives of researchers within a newly formed research institute about principles and practices of research codesign in the context of their previous and current projects. We also investigated their perceptions of institution-level enablers and barriers to codesign. University based researchers are our primary focus here and we intend to consult other stakeholders in future work.

**Methods:**

Using an interview guide informed by exploratory work and a scoping review of the literature, we conducted 15 individual interviews with Caring Futures Institute (CFI) leaders and researchers at different career stages working across multiple areas of health, care, and social research. Qualitative thematic analysis was conducted.

**Results:**

The researchers we interviewed were involved in projects ranging from large nationally funded projects to small studies funded by the university or PhD projects. Research codesign activities were generally part of larger researcher-led projects but there were a few examples of community-led projects. There was agreement amongst participants on the principles and perceived benefits of research codesign such as partnership, co-learning, and power sharing. Less agreement was found regarding the definition of research codesign and best terminology to be used. Themes reflecting the success of research codesign included pre-existing community relationships, communication skills, knowledge, and training on codesign, balancing power relationships, use of external facilitators, and adequacy of funding, time, and resources.

**Conclusions:**

The study reaffirmed the complexity of research codesign from researchers’ perspectives and identified areas of potential action that may be beneficial for university based research institutions in building codesign skills, capacity and culture for example training, peer learning and funding support. Implications for practice improvement centre on a dual strategy of building practical capacity in researchers and integrating institutional dimensions (such as governance and leadership) into codesign frameworks. This can help to ensure research codesign is integrated into organisational culture and through the work of individual researchers.

**Supplementary Information:**

The online version contains supplementary material available at 10.1186/s40900-022-00392-4.

## Plain English summary

Involving people and groups with lived experience in research is important to ensure that research is useful and makes real changes in peoples’ lives. Codesign centres on researching with people rather than about people. For true engagement and codesign to occur, university based researchers need to understand why codesign is valuable and need to be trained and supported in using effective methods for the individuals and groups they work with. University based research institutes with missions to improve peoples’ experiences of services need to embrace principles and practices of codesign and support their researchers to achieve this. They need to make sure there are appropriate governance structures and support systems that encourage people to codesign in their research. This study focussed on university based researchers as a key stakeholder group for effective codesign. These researchers work in an Australian research institute (Flinders Caring Futures Institute). They shared information about the kinds of codesign they did in their research projects, and the things that helped and other things that made it hard to utilise codesign in their research. We also asked the researchers about how research institutions could better support codesign practices. Researchers in our study shared their views that codesign is a complex process. Building relationships and trust as part of research codesign needs time and resources. Researchers had ideas about how to improve codesign as a practice in research institutes. These include providing formal and informal training, opportunities to share experiences, peer support and learning, making sure there are community representatives involved in the leadership of research organisations, and increased funding support for codesign.

**Supplementary Information:**

The online version contains supplementary material available at 10.1186/s40900-022-00392-4.

## Background

The term *codesign* is broadly defined as a process of involving all ‘stakeholders’ which includes ‘consumers’, ‘communities’, and ‘end-users’ in the planning and design, implementation and evaluation of products, services and research to combine lived experiences and professional expertise [1, 2]. The concept of codesign is underpinned by democratisation theories and rights-based approaches where communities and individuals, and sometimes organisations, have the right to have an input into services and research on their conditions [3]. Codesign is seen as a mechanism for empowering communities and individuals to develop their own ideas, knowledge and skills to address their problems [2, 3]. Principles and values underpinning codesign include mutual respect, participative process, inclusiveness, flexibility, fairness of opportunity and accountability [1, 4–6]. Other issues such as data and cultural sovereignty may also enter into some discussions around codesign [7, 8].

The current study reports on qualitative research with one stakeholder group, university based researchers, to better understand how they view and participate in codesign activities. We acknowledge that there are multiple stakeholder groups involved in effective codesign and that there are limitations in only presenting the views of one stakeholder group. However, as a newly developing research organisation, we felt a reflective paper focused on the diverse perspectives of university based researchers from different disciplinary backgrounds and career stages was a systematic first step in understanding our vantage point and relative contribution. We acknowledge that some university based researchers may also be end-users of research although we didn’t explore that in this study.

In the academic literature, *research codesign* in health contexts is defined as “the meaningful involvement of research users during the planning phase of a research project, where meaningful involvement is taken to refer to participation in an explicitly described, defined and auditable role or task necessary to the planning and/or conduct of health research” (p. 3) [9]. There is a lack of consistent conceptualisation and standardised interdisciplinary terminology for research codesign in the literature [10, 11]. Codesign is used, however, as an umbrella concept with a wide range of terminologies and approaches used to demonstrate broad engagement in research processes that extends beyond the researcher versus researched divide. Examples include ‘patient involvement’, ‘stakeholder engagement’, ‘patient-centred research’, ‘collaborative research’, and ‘community-based participatory research’ [9]. Put simply, research codesign is about conducting research *with* people rather than *about* people.

Consumer and community involvement in research is strongly encouraged by the National Health and Medical Research Council (NHMRC) funding body in Australia [5] and emphasised in the Australian Code for the Responsible Conduct of Research: “appropriate consumer involvement in research should be encouraged and facilitated by research institutions and researchers” (section 1.5) [12]. Intensity of involvement ranges from relatively passive to highly active involvement of communities, individuals, and organisations in research [1, 9]. Research codesign approaches also vary in the research stage at which communities provide input (e.g., community involvement in the initial design and conduct of research [13] in contrast to engagement in the interpretation and dissemination of research findings) [14]. The most common codesign tasks reported in the literature are seeking public or community inputs in research agenda and priority setting [15, 16], reviewing research proposals [17], and contributing to research development and validation [18] via community advisory committees/boards and individual or group interviews and meetings [15]. Other activities less frequently used are citizens’ juries, workshops, forums, nominal group techniques and Delphi techniques [19].

Benefits identified from research codesign include increased acceptability and applicability of research topics, questions and materials and translation of evidence that can advance policy and practice [14, 20], enhancing knowledge, skills, confidence, and the sense of accomplishment and empowerment amongst end-users [18, 21]. Another benefit is improved access to research participants, especially people who are usually under-researched or less likely to be approached in research, also improving research response rates of interventions [18]. Nevertheless, there are challenges reported in the academic literature associated with research codesign. These include increased time to undertake the research and the need for additional financial resources [15, 20], inadequate transfer of power, tensions between researchers and end-users [18], and ensuring research rigour while incorporating end-users’ preferences [22].

Several frameworks and toolkits have been developed to guide researchers seeking to use codesign approaches. The UK Economic and Social Research Council developed the Research Impact Toolkit which has a strong emphasis on collaborative research involving users at all stages of the research to enhance research impact, academically and socially [23]. The Australian Clinical Trials Alliance’s Involvement and Engagement Toolkit [24] provides an interactive map on how researchers and research organisations can involve patients in different phases of clinical research. Shippee et al., through converging various frameworks of patient and service user engagement from 37 sources, developed a synthesized framework that comprises three broad phases of research (preparatory, execution and translational) and presents specific activities under each phase ranging from passive engagement (e.g., consultations) to a more engaged approach (e.g., lay and public controlled) in a comprehensive and logical way [25]. More recently, Greenhalgh et al. reviewed 65 health-related research codesign frameworks and grouped them into five broader categories (p. 788-789) [26]:

- *power-focused*, exploring and overcoming researcher-lay power imbalance
- *priority-setting*, involving patients and lay people in setting research priorities
- *study-focused*, increasing recruitment and retention mainly applied to clinical trials
- *report-focused*, guiding write up and critical appraisal of research reports
- *partnership focused*, increasing transparency and public accountability in research-lay collaboration

There is little evidence in the literature about the evaluation of codesign approaches. As far as we are aware, no studies have compared the effectiveness, outcomes and/or cost-effectiveness of research codesign approaches against traditional research processes [9, 11]. Rather, reports of codesign evaluations are limited to researchers and end-users’ perceptions about the benefits and impact of codesign on research process, and the time and resources required (short-term evaluation via qualitative studies) [9].

Little is known about how research institutes adopt a coherent approach to codesign and the way that researchers view and employ codesign in their daily practice. This is particularly important in the context of multidisciplinary research institutes comprising researchers from diverse disciplinary backgrounds, different career stages, and a wide range of population groups as research participants and collaborators.

In summary, there is a knowledge gap in university based researchers’ knowledge and practice around codesign and the role of research institutes to build capacity and capability in research codesign through governance, methodology and capacity building initiatives. This is important to maximise the impact of research institutes and researchers, particularly when aiming to change policy or practice to improve outcomes for communities and individuals.

It is in this context that this study aimed to investigate how university based researchers within a newly formed research institute understand and implement the principles and practices of research codesign within the context of previous and current projects. The study explored the experiences of individual researchers, and their perceptions of institution-level enablers and barriers to research codesign. The intent was to inform future strategy, governance and support that research institutes can offer to enhance research engagement and impact. At this early stage in our development, there was no public or community involvement in the study. We acknowledge the importance of this missing perspective and plan further research to build on our preliminary study once our own context is mapped and understood.

### Study context: Flinders Caring Futures Institute

This project was conducted in the setting of a new research institute: the ‘Flinders Caring Futures Institute (CFI)’. CFI was established in 2019 in the College of Nursing and Health Sciences, Flinders University of South Australia. The institute is Australia’s first fully dedicated research centre for the study of self-care and caring solutions and comprises multidisciplinary research teams which work in partnership with health and community services professionals, service providers, patients, service users, carers, community members, funders and policy makers in order to improve care experiences and services (https://www.flinders.edu.au/caring-futures-institute). Partnerships with stakeholders is a central building block of the CFI both in governance, structure, and activity. The institute is organised across four main themes: researching care across the lifespan around health and care promotion (Better Lives); supporting marginalised and disadvantaged populations (Better Communities); exploring caring innovations and interventions (Better Care) and health and care service research (Better Systems). Each of these themes is supported by teams with knowledge translation and implementation science, technology, health and social care economics and methodological expertise. In turn, researchers bring expertise from a diverse range of disciplines into a number of areas including, but not limited to, a healthy start to life, integrated care, disability and social inclusion, ageing and aged care and cardiovascular care innovation.

Stakeholders’ engagement, collaboration and codesign are underpinning principles of CFI’s establishment. Codesign and participatory research approaches are recognised as a methodological building block for the whole institute as part of an integrated knowledge translation philosophy. Given the eclectic nature of CFI researchers and the breadth of themes and topics they are involved with, it is critical to understand the expertise and experience of these researchers and their teams around codesign. Using CFI as an example, this study will provide insights about complexities in research codesign with the aim of improving governance processes and codesign support systems within research institutes that share similar objectives (i.e., a focus on research impact to improve policy or practice and the lives of individuals and communities).

## Methods

This study received approval from the Flinders University Human Research Ethics Committee and participants were assured about the voluntary nature of participation, and confidentiality issues. Invitations to take part in the project were sent to potential participants along with an information sheet and consent form. Participants were selected purposefully to include researchers from different disciplines and career stages and those whose research had potentially involved stronger community involvement. An initial list of potential participants who worked in the CFI was developed. Convenience sampling was used for initial interviews. Additionally, snowball sampling was used when interviewees suggested a researcher in their discipline who they believed to have experience of working with different population groups in their research. Of 16 people invited, 15 agreed to take part. We conducted semi-structured individual interviews with study participants. Interviewees represented different career stages (4 professors, 10 early- and mid-career researchers, and 1 PhD student) and different disciplines. Thirteen out of 15 participants were females, reflecting CFI’s broader demography.

The individuals we interviewed were involved in research projects that varied in scale, disciplinary focus, target population, and the source and size of supporting funds. These ranged from larger projects funded by national funding bodies (e.g., Australian National Health and Medical Research Council; Australian Research Council) to medium and small-scale projects funded by government (e.g., South Australian Department of Health), not-for-profit organisations, professional associations, Flinders University internal grants, and PhD projects across different health and social research areas. Project funding ranged from less than $10,000 to over a million Australian dollars.

We used inductive and deductive approaches. The interview schedule included questions about codesign processes. We also sought participants’ understanding of research codesign, different terminologies used, experiences of undertaking codesign activities, factors which enabled or constrained the success of their codesign approaches and any challenges that they may have encountered. Participants were also asked to comment on strategies which could be implemented to enhance institutional support on research codesign (See Additional file 1 for interview questions).

Interviews were conducted between March and April 2021 with each interview taking approximately 40–60 min. Interviews were audio-recorded, transcribed and de-identified for further analysis. Qualitative thematic analysis was used with assistance from NVivo-12 software for data management and coding. A coding structure was developed based on deductive codes from the research questions. We also generated codes inductively to capture additional concepts emerged from interviews. The coding structure was reviewed and refined to group codes that were related to similar themes. Interviews were coded and analysed by the first author (SJ). Themes drawn from the coded data were discussed on multiple occasions with the corresponding author and as a team. A number of participants provided project materials (e.g., grant proposal) as evidence of codesign activities. These materials were used to confirm interview data where possible.

## Results

Participants shared their perceptions about research codesign and discussed examples of codesign activities from one or several projects in which they have been involved. Several themes were drawn from the analysed data, reported below. These are presented under six main themes: 1) understanding of research codesign; 2) range and breadth of codesign approaches; 3) factors enabling research codesign; 4) barriers to research codesign; 5) evaluating the effectiveness and efficiency of research codesign; and 6) institutional policy and funding to support research codesign.

### Understanding of research codesign

#### Definition and terminology used

Participants provided mixed views about the concept and terminologies used to best describe research codesign. Whilst some viewed codesign as an umbrella term to describe stakeholders’ involvement in research, others made a clear distinction between different terminologies and underpinning ideas. One participant noted:

I use coproduction and think about codesign as fitting inside coproduction. I think of them as fairly specific concepts, I don’t think of them as interchangeable, they’re contested concepts. (ID#3)

In contrast, another participant placed an emphasis on the ‘design’ element in research projects:

I prefer ‘codesign’ because it has that open-ended ‘we’re designing something’ whereas coproduction kind of implies the production of a thing…(ID#4)

Nevertheless, there was a theme reflecting consistent values and principles underpinning research codesign. Participants frequently mentioned phrases such as *‘reciprocal relationships’*, *‘co-learning process’*, *‘power sharing’*, *‘community control and ownership’*, and *‘collaboration and engagement’* while defining research codesign.

Although semantics seemed to be important in better understanding of the concept of codesign for some researchers, others believed that the actual approach one takes in practice mattered more than a focus on language and terminology.

I don’t think language matters that much… one came from one field, and one came from another but both fields were trying to achieve the same thing, which was working with groups outside of academia from the beginning. (ID#8)

Several participants further detailed research codesign as a human right-based ideology, a philosophy, and a value system that considers meaningful involvement of stakeholders and communities in any research about people and their conditions as people’s right. They viewed codesign beyond categorisation as solely a research methodology or set of activities and saw it as an approach to build relationships, empower communities, and to make real changes and long-term solutions to existing problems.

One of the things is how you actually move from applying codesign as a technique to embedding it as a practice…I think we tend to think about codesign as something to tick off for your funding application or a process to do research in the approved way, rather than a way to extend and learn. It’s a philosophy really… (ID#3)

Another participant noted:

I guess codesign describes a value system and a set of principles and a way of engaging with people rather than a specific methodology or specific type of outcome. (ID#4)

#### Codesign versus consultation

Participants generally agreed on the inclusive nature of a ‘true’ codesign in clarifying problems and/or developing solutions and how it differs from consultation where stakeholders’ views on specific aspects of research are sought in a highly researcher-controlled process.

Sometimes codesign is actually just consultation, we have to look really critically at what is happening in codesign and whether influence and authority is really vested in people who are involved in research activities or whether we’re just consulting with people... there’s nothing wrong with consultation, but we should just call it what it is (ID#3)

Comment from another participant supported the notion that consultation does not necessarily fulfill requirements for a good codesign process.

We see a lot that people create their own stuff, and then they go to the consumer and ask, ‘what do you think about this?’ it’s more like consultation. That’s the process of ticking a box and that’s well-defined by consumers as tokenistic. (ID#5)

#### Advantages and disadvantages

Whilst acknowledging different research approaches, participants stressed that benefits can be gained from involving stakeholders in research. Examples included research quality, acceptability, and impact, as well as minimising the divide between researchers and research-users, mutual learning, and community empowerment.

It’s [codesign process] a positive thing because - researchers and clinicians, sometimes there’s a divide between the two, so it helps to create that sense that we’re all working together. (ID#1)

Participants expressed how employing codesign approaches mutually benefit researchers and stakeholders through learning and empowering both groups:

It’s two-way learning, we’re also learning from the individuals with [a health condition]. So, that’s a critical element of codesign—I learn from you, you learn from me. (ID#15)

A few participants raised issues around complexity of stakeholders’ engagement and meeting their expectations, as well as additional time and resources required to do so.

When you do codesign, you factor in other people and what they care about. And sometimes they want to scale it, but I’m like ‘I don’t know if we collected data for that or have resources, I don’t know how to do that’. And saying no to them also isn’t an option. So, we’re really managing that tension now, we don’t want to let them down. (ID#8)

### Range and breadth of codesign approaches

Participants commented on the range of stakeholders that need to be involved and engagement methodologies as below:

#### Who to engage with and when

Codesign was seen as a complex process involving multiple stakeholders, including community representatives, service providers, practitioners, service planners and policy makers. Participants, however, shared a common view that groups to engage with and the research stage at which engagement needs to occur vary depending on the nature of the project, research aims and expected outcomes:

It depends on the problem and the issue that you identify who and at what point those key groups you want to engage…everyone is potentially a stakeholder; it depends on their interest in the problem. (ID#11)

Some participants provided examples of where a specific stakeholder group were more relevant to their projects.

If it’s an intervention project and one of the aims is to provide the evidence to inform the policy and the resource development… we definitely need to engage the policy makers into the project to provide the feedback throughout the project. (ID#10)

Early- to mid-career researchers reported less experience of and skills to engage with wider stakeholders, especially policy makers.

I haven’t had much to do with the bigger picture, bringing in different kinds of stakeholders. For me, it’s really been working alongside people with [the health condition]. (ID#15)

Engagement with people not directly linked to the research was also felt crucial to ensure various perspectives are included in the topic of interest:

In the context of some projects, you really need to speak with all stakeholders, and not just stakeholders that you view as being relevant, you need to be aware of people who consider themselves a stakeholder even if you don’t think they are. Perceived stakeholders…’ (ID#8)

Nevertheless, availability of stakeholders and feasibility of engagement were seen as factors limiting the ability and thus scope and range of engagement.

Whilst it’s wonderful to involve everybody, feasibly it’s not always possible. It’s certainly not possible with the size of the grants that tend to go along with this sort of thing.’ (ID#6)

#### Codesign activities, processes, and methods

Codesign activities described by participants ranged from being only one small element of a large researcher-led project to activities that were entirely led and owned by the community group of interest with the researcher playing a facilitator role. In most cases, codesign was used as a methodology to identify a problem or find/test a solution concerning an issue of interest.

Our project differed from a codesign project where you’re starting with just an idea or something that’s quite undeveloped. We started with something that’s already quite established… so I feel like we were a few steps into that process of defining what the project is. (ID#1)

A few participants provided examples of using codesign approaches at earlier stages of research (prior to writing grant applications) to develop research questions or through the whole process of research design, implementation and dissemination.

We invited consumer representatives into the codesign group and developed the draft of the proposal before we submitted to the funding body. (ID#10)

Another participant used the term ‘community-led’ project where their community group of interest were involved in the whole research process.

This project was mostly consumer led... involving consumers since developing the research question, through ethics application, designing questionnaire until writing the paper…we have presented in several conferences peer-to-peer… she led the presentation, and we were supporting her. (ID#5)

Individual interviews, surveys, forums, focus groups and workshops with consumers/patients, clinician and/or other stakeholders, and community representatives in research advisory committees were most frequently reported as codesign methodologies. A few participants, however, provided examples of more innovative engagement methodologies:

We ran workshops and used lots of photos, pictures, and drawings where people could express what their problems were. (ID#9)

### Factors enabling research codesign

Participants shared their experiences of factors that enabled research codesign in their projects. These include:

#### Pre-existing relationships with communities and other stakeholders

Pre-existing relationships with communities and other stakeholders was reported as crucial in initiating and maintaining engagement in research.

The work is possible if you have those relationships already in place. You have to keep those relationships alive in order to keep people employed and to keep the work happening. (ID#3)

#### Communication skills and capabilities

Good communication skills that embrace mutual respect, inclusion and trust were seen as critical in building and maintaining relationships with stakeholders. For those participants with a clinical background, being trained in communication skills as part of their educational training has been an enabler to better engage with communities in research.

As a clinician you have to establish relationships quite quickly…you have to be respectful, you have to be friendly, it has to be a nice experience for patients. So, I think some of this is just interrelationship skills that you can practise in research too. (ID#2)

#### Use of external facilitators or community coordinators

Some participants provided examples of how the use of an external facilitator with a special set of engagement skills assisted in codesign processes and balancing power dynamics in groups particularly when working with diverse population groups.

The people we worked with [external facilitators] were extremely skilled in how they facilitated it [workshops]… and making sure people felt like they would contribute. So, for me they were the positives. (ID#9)

Another participant reaffirmed this point:

I’m of the opinion that it’s always better to have someone external to come in, because then I’m sitting there as part of the group and I’m not up there presenting a lecture. (ID#4)

Others noted the important role that community coordinators or champions have played in bridging communities to the research team and building trust.

We had community connections, people who were specifically working on those community connections who were ingrained in the community, we had people working across building those bridges…so that worked really well. (ID#12)

### Barriers to research codesign

#### Codesign is a complex, non-linear process

Overall, there was a common theme highlighting that research codesign is a complex and time-consuming process which does not fit easily into existing linear frameworks. The complexities around building relationships and understanding stakeholders’ roles and expectations usually make it difficult to implement a codesign approach within the context of most research projects.

So much easier said than done, though. Whose boss is going to let you… it’s time-consuming, before you even have funding to support the project… you can have all these frameworks but in reality, it’s never going to be linear… (ID#8)

One participant commented on the way that researchers normally view different stages of the research which might be different to stakeholders who are involved or impacted by research:

We often think of research as compartmentalised- it’s this stage or it’s that stage and then we move onto the next stage. But for the people who this research will impact, they don’t see any of it unless they’re part of the whole journey. (ID#12)

#### Limited funding, time, and resources

While some projects had funding allocated for codesign activities such as consumer workshops, in most cases, the lack of funding and resources was a major limitation. This was especially so when funding was required at earlier stages of the research (e.g., in developing research questions or codesigning research methodologies). It was felt that external funding bodies (especially large national funding organisations) are not willing to fund research projects that propose ‘vague’ interventions that rely on the outcome of codesign processes.

You’re just trying to sell what you’re going to do when there’s a lot of vagueness, because you are saying ‘we’ll figure it out as we go’, and that balance of trying to look like you know what you’re doing verses the scope for things to go where they naturally go. (ID#8)

Acknowledging contributions to research by reimbursing individuals and groups for their time and intellectual inputs was other important issue that requires funding not normally supported by funding bodies.

That’s a really critical element, because the co-researchers contribute voluntarily- which doesn’t sit right morally, ethically or philosophically- so now we’ve got this underlying principle. If we can’t get funds, we probably wouldn’t engage the person and you couldn’t do a codesign project. (ID#15)

Most participants felt that the time spent on relationship building and engagement is not always acknowledged by universities and not included in key performance indicators which makes it difficult for researchers.

It [time spent on community engagement] has definitely affected my track record because we’ve always prioritised the accessible outputs and academic papers. But I don’t regret… because I got some amazing relationships and collaborations out of it and that’s been very rewarding. (ID#3)

#### Poor codesign knowledge and training

Poor education and training around research codesign concepts and principles, guiding frameworks, and methodologies was seen as a barrier. Most participants, particularly early- to mid-career researchers, expressed concern about their poor knowledge in this area and felt that training provided as part of academic courses or career development processes had not equipped them appropriately with the skills required to employ codesign approaches and to manage associated challenges. Similarly, it was felt that communities are not trained and supported enough about their roles and expectations regarding research and ways that they can best participate and contribute.

Many participants stated that they did not have any training on codesign activities prior to the commencement of their research project(s). Furthermore, research workload and timelines did not allow researchers to seek and receive training they required during the project.

…not having any formal training to understand what framework we should use. I would say it is a barrier because it takes more time to get your head around what you’re doing…(ID#1)

An early-career researcher noted poor codesign knowledge and skills as a barrier to employ innovative engagement methodologies:

We’re not trained in that [co-design], but we should be. All we’re good at is interviewing or surveys, which only is good if the person has something to say, and if they don’t know what to say then you’re like ‘oh ok, next question’. (ID#8)

#### Power imbalance

Participants highlighted power imbalances between researchers, communities, and other stakeholders as one of the key factors hindering the level and success of engagement, also one of the most difficult challenges to deal with.

We’re very hierarchical. Health professionals hold the information…And to lose that and to come in with a different power base, a lot of people find it very threatening, but I think that’s what a good codesign would do. (ID#2)

Another participant highlighted the challenges associated with power dynamics while working with stakeholders.

Different power dynamics in a group is challenging. We often talk about building from being a novice facilitator to being an expert and you’ve really got to mentor people once they first do it. You can’t just send them out into the world to do this work. (ID#9)

### Evaluating the effectiveness and efficiency of research codesign

#### Lack of codesign evaluation

Very few participants reported strategies to evaluate the effectiveness of codesign activities in their projects. It was evident from the interviews that evaluation was often limited to an assessment of community perspectives on workshops or project outcome measures rather than longer term efficacy of codesign approaches.

I think there’s two aspects to evaluation, is that process evaluation in terms of the actual research process, and then evaluation of whatever was implemented and the outcomes around that.…it’s not something I’ve ever followed up on in terms of closing that loop, But I think that’s a really important aspect of the whole process. (ID#7)

There were however a few examples of more comprehensive evaluation of codesign activities. One participant stated:

I created a methodology to use mix methods, qualify and quantify not only the codesign process, but also the products that come from that process. So that we qualify and quantify the impact of them, and you can compare across processes and products in a standardised manner. (ID#5)

Another example from a PhD project has a focus on codesign evaluation:

Well, the project is formally evaluating the entire process. So, there would be that insight into ‘how did it go? What was it like?’ The PhD student is collecting that data along the way, she’s got surveys, questionnaires, interviews, field notes. And she’ll actually produce that output in terms of evaluating that codesign research process. (ID#7)

### Policy and funding to support research codesign

We asked participants about their views on institutional support and strategies that may improve the understanding and implementation of codesign approaches at an individual level but also enhance codesign culture and support at higher institutional levels. The key strategies suggested are:

#### Building codesign skills and capacity

The inclusion of engagement and codesign concepts in undergraduate and postgraduate curricula, as well as training workshops for established researchers were frequently mentioned as strategies that research institutions such as the Caring Futures Institute could take into consideration.

I really didn’t know anything about it [codesign] until I had to go looking, perhaps something at undergrad course or at Master level or when people do honours…I also think having some workshops or training for researchers who are wanting to undertake that as a form of approach to their study, so that people have got a bit more knowledge and feel a bit more confident and empowered. (ID#1)

One participant pointed out the importance of having a definition and different ways of doing research codesign within the institute to guide researchers.

First step is to do this sort of benchmarking to be able to define it and to have the leadership of CFI [Caring Futures Institute] come together and say ‘this is our definition of codesign, this is why we do it and this is how we do it and these are the products that we expect to come out of this’. Then you would develop your training materials. (ID#11)

#### Learning from experience

Having researchers who are highly experienced in research engagement methodologies within the institute, participants strongly supported the idea of putting formal structures in place that enable researchers to share experiences and learn from each other. The opportunity to discuss innovative methodologies, ways to manage potential challenges, and sharing examples of successful grant proposals, codesign projects and resources was highly recommended.

I think seeing other people doing it and learning the tips on how to do it and how to do it well helps. I don’t think books and websites help. I think seeing the successful projects and seeing what comes of it and hearing the stories. (ID#2)

If we set some examples and guidelines. You know, examples of grants that have been successful, so we know what words to use, learning from each other… we should just have this database somewhere. (ID#6)

Mentoring programs were felt helpful in supporting researchers:

We would maybe monthly have mentoring meetings, where they would come back and say, ‘oh I went there and did this and it went absolutely wrong’, or ‘something happened and I didn’t know how to handle it’ and then you discuss it and say ‘well, what are the strategies that might have worked’. Again, it’s very much based on the action learning principles. (ID#9)

#### Stakeholders’ representation in governance structures

As a research institution, having ongoing relationships with communities was seen as critical. Some participants found it difficult to initiate new relationships. Strengthening community membership in organisational governance and leadership structures were suggested as strategies that provide opportunities for stronger links and early engagement with consumer organisations and individuals.

Maybe [community representatives] being embedded as part of the institute. So, I think that can really help. How can that be managed in a more systematic way which not only helps the community, but it also helps us as well. I think for me that’s the toughest part- I don’t really know where to start. (ID#7)

#### Codesign funding support

Participants highlighted the importance of institutional support in seeding research codesign activities through funding. Several participants felt that institutional support, for example, through funding to recognise time and contributions of stakeholders in projects, would encourage codesign, and accelerate opportunities for ECR and PhD students.

I think some support and grants to assist it. … but if you’re doing codesign, ‘we have some funds, how many participants are you going to have? What’s your project? You just apply and you get $50 per participant and that will help you’. (ID#6)

Funding opportunities to recognise stakeholders time and contribution as coresearchers were highlighted:

Having those opportunities for grants where you can use to pay a coresearcher. So, resourcing it- funding it, it’s about trying to provide opportunities. So, perhaps when there is a grant round coming up, we could consider some that are inclusive. (ID#15) 

Table 1 summarisied key findings from the study and proposed actions recommended by the study participants.

**Table 1:** Summary table

Themes/subthemes	Key findings	Proposed actions for research institutions and funders
*Understanding of research codesign* Definition and terminologies Underpinning values Codesign vs consultation Advantages and disadvantages	Mixed views on how to define and best terminology to use Agreement on underpinning principles of mutual respect, empowerment, power sharing and engagement Perceived benefits for both researchers and communities including research quality and impact Complexity of codesign process and the need for additional time and resources	*Capacity building* Assessment of knowledge gaps/educational needs Teaching codesign topics and examples to undergraduate and postgraduate students Training workshops for researchers Mentoring program Developing guidelines Capacity building for other stakeholders including consumers and communities *Experience sharing* Formal structures in place to share experiences/stories and learn from each other Discussing innovative codesign methodologies, how to manage potential challenges Sharing examples of successful grant proposals, codesign projects and resources Peer support program within, and between, institutions *Stakeholders’ representation in institutional governance structure* Building formal and informal relationships with different stakeholder groups and organisations through projects and governance structure Strengthening external and community membership *Funding support* Provision of seeding funding for codesign activities Recognising/reimbursing stakeholders for their time and intellectual inputs into research *Institutional culture supportive of research codesign* Encouraging and rewarding codesign projects Inclusion of codesign activities in research performance metrics
*Range and breadth of codesign approaches* Who to engage with and when Activities, processes and methods	Research involvement and engagement may vary depending on the nature of the project, research aims and expected outcomes Need to gain skills and experience around codesign particularly for early career researchers Availability of stakeholders and feasibility of engagement may limit ability and scope of engagement Codesign activities range widely: from small element of a larger researcher-led project to a project entirely led by communities and facilitated by researcher	
*Factors enabling codesign*	Pre-existing relationships with stakeholders Communication skills and capabilities External facilitators and community coordinators Community coordinators or champions	
*Barriers to research codesign*	Complexity, non-linear processes Poor understanding of stakeholders’ roles and expectations Poor codesign knowledge and training Power imbalances Limited funding, time and resources from institutions and external research funding bodies	
*Effectiveness and efficiency of research codesign*	Evaluation usually limited to an assessment of community views on projects or research findings Complexity of evaluating codesign approach as a philosophy and value-based way of thinking and researching	

## Discussion

Given the growing attention towards public involvement in research to maximise research impact and facilitate the translation of evidence into policies and practice [17, 26, 27], there is an urgent need to identify existing challenges and ways to enhance research codesign support. Universities and research institutes play a critical role in training and mentoring current and future researchers to understand concepts around research codesign and learn strategies to implement and manage its processes.

Our study exploring the perspectives of university based researchers demonstrated a lack of consistency in the way research codesign is defined and understood, and in approaches to implement codesign. Language and terminologies are to be context specific and may vary according to the nature of the project and the preferences and priorities of end users [26]. However, it is crucial that underlying this diversity is a common understanding of the key principles, processes, and practices that underpin research codesign. The literature identifies the value of codesign frameworks in providing a scaffold for researchers and organisations to approach codesign [25, 26]. Research institutes are well-placed to provide a supportive environment to encourage, build capacity and reward research codesign culture and practice. Existing challenges and potential opportunities are discussed here.

Researchers who participated in our study reaffirmed the complexity and non-linear nature of codesign. This means that although research institutes play a critical role in improving process knowledge and skills and creating a research culture supportive of codesign, a single and one-size-fits-all approach may not be applicable to all research areas and contexts. As noted by Greenhalgh et al. there is limited transferability of existing guides across various contexts, rather they need to be adapted and combined in a ‘locally generated codesign activity’ [26].

Findings from our study provide insights on the key challenges researchers face and institutional actions that could improve research codesign practice and culture. The need for formal or informal training to address codesign knowledge gaps was strongly recommended by participants. This seems to be a particular issue of concern for early- and mid-career researchers. Assessment of educational needs at the institutional level and inclusion of codesign training in broader capacity building strategies to address various needs must be considered [28]. Learning is also gained by experience sharing and peer support within and between institutions and was highlighted in our study. Some universities already have strategies in place that can be adapted, for example codesign living labs to bring together researchers, industry, policy actors and people with lived experience to brainstorm and codesign research ideas, and undergraduate topics and short courses on codesign [29, 30].

There is a need to build capacity in the area of codesign beyond individual projects and towards institution-level strategies and culture which supports and shapes the ways their research activities are developed, implemented and evaluated. Our study confirmed the importance of research governance and leadership, infrastructure, and funding within research institutes (but also research funding bodies) to recognise and support the extra time and resources required for codesign. Provision of such support systems within the funding constraints and performance metrics of the higher education environment may be challenging [31]. However, to achieve institutional goals regarding engagement and impact, research institutes such as the CFI need to activate flexible measures and incentive systems to enable researchers to engage with stakeholders in timely and meaningful ways.

Finally, monitoring progress and evaluating outcomes is important. Our study supported the existing gap in the literature about codesign evaluation to examine the extent to which codesign approaches actually improve end-users’ health or wellbeing outcomes or the research quality and rigour compared to traditional research processes [9]. Evaluation activities described by participants in our study mainly focused on consumers’ perceptions of the benefits of the codesign process. Further research is recommended to better evaluate the effectiveness and cost-effectiveness of research codesign. Codesign is a philosophy and value-based way of thinking and researching, and not purely a research method. Broadening our approach to measurement is not simply a matter of adding codesign to existing approaches to research evaluation methods. Developing a theory of change that links consumer and research outcomes to broader health and social impacts is suggested as an approach to address the challenge of real-world impact of research codesign [9, 28]. A study protocol published by L’Esperance et al. initiating the process to develop a Canadian evaluation framework for patient and public engagement in research is promising as a guide to researchers in other settings [32]. At the institutional level, training and supporting evaluation approaches which include codesign is crucial to demonstrate the effectiveness and efficiency of codesign approach in research.

### Study strengths and limitations

To our knowledge, this is the first study investigating the views of university based researchers from different disciplinary backgrounds and career stages about their understanding of and experiences with co-design, and their perceptions about organisational opportunities and gaps, and support systems that are required to ensure codesign is embedded into research culture and practice.

The study, however, has some limitations. First, we point to the obvious limitation that the study we describe in this paper does not reflect codesign or coproduction in its methods. Our study only included university based researchers as one stakeholder group in codesign process. Other stakeholders, for example, end-users, community groups participating in research, collaborating organisations, and policy actors also play a critical role in the process of research design, implementation and translation. The study reported here will be the basis for future studies to explore the viewpoints and experiences of other stakeholders in the research codesign process and the role that they can play in institutional governance structure. Engaging with other stakeholders is particularly crucial to further explore issues in relation to power balance at all levels of codesign construct and its underlying philosophy. Moreover, an exploration of training needs for stakeholders outside academia and the development of appropriate capacity building approaches is important to increase all stakeholders’ knowledge and contribution to codesign processes.

Second, we used the term ‘research codesign’ because it is most commonly used in the literature. However, we acknowledge that, for some, the term ‘codesign’ may not capture a research process that involves all stakeholders in design, implementation, production, and evaluation of research projects, and translation to policy. Third, a relatively low number of participants (n = 15) in our study limits the saturation and generalisability of findings. This study was a small exploratory project and participants were mainly recruited based on a convenience sampling approach. Comparative studies involving a greater number of university based researchers from different institutes and disciplines will be useful to provide comprehensive information about strategies that may work (or not work) as well as organisational factors, leadership and infrastructure that enable or constrain research codesign.

## Conclusions

Research codesign increases research impact and the successful translation of evidence into policy and practice. This study focussed on one stakeholder group in the codesign process, university based researchers. Based on researchers’ perspectives, we identified areas of potential action in research institutes that may be beneficial for future planning and investment around research codesign skills and knowledge, capacity and culture as well as funding support. Implications for practice improvement centre on a dual strategy of building practical capacity in researchers and integrating institutional dimensions (such as governance and leadership) into codesign frameworks to ensure codesign is embedded into organisational culture as well as the work of individual researchers.

## Supplementary Information

**Additional file 1.** Interview questions.

## Data Availability

Interview data for the current study are not publicly available due to participant confidentiality.

## Abbreviations

NHMRC: National Health and Medical Research Council
CFI: Caring Futures Institute

## Declarations

### Authors’ information

SJ is a research fellow in the College of Nursing and Health Sciences. SR is a Professor in Disability and Community Inclusion and was lead of the Better Communities theme at Caring Futures Institute. AK is the Vice President and Executive Dean of the College of Nursing and Health Sciences, Foundation Director and lead of the Knowledge Translation theme at Caring Futures Institute. JA is Dean of Research in the College of Nursing and Health Sciences and lead for the Methodological Innovation theme at Caring Futures Institute.
