# Supplementary material for: Embedding research codesign knowledge and practice: Learnings from researchers in a new research institute in Australia
Source: Res Involv Engagem. 2022 Dec 7;8:71. doi: 10.1186/s40900-022-00392-4 (PMC9730560; doi:10.1186/s40900-022-00392-4)
Supplement: Supplementary file 1 — Additional file 1. Interview questions. [file 40900_2022_392_MOESM1_ESM.pdf]

Interview questions

1. I would like to start with a broader question and ask how would you define research co-design?

[Probe: community and end-user's involvement in different stages of the research versus broader stakeholders such as health providers, policy makers, etc etc)

2. Various terminologies are used for research co-design. For example 'Participatory action research', 'Community engagement', 'Stakeholder engagement and involvement', 'Co-production', 'Inclusive health research'.
   Which of these terminologies do you normally use in your research or apply better to your research and why?
   Anything you would like to talk about history of research co-design and how it has changed (or attracted more interest) in research?

3. Thinking of one or more of your research projects, can you tell me a bit about the nature and scale of those project(s)? you can talk about one or more projects
   - What is the project's scale? (duration, funding, team, etc)
   - What population group(s) are targeted in your projects?
   - What is the area or discipline?
   - What other stakeholders are involvement in your project?
   - At which stage of the research have you involved communities?

4. Can you share with us the range and type of co-design activities and resources that you have employed in the study?
   - At which stage of the research (developing research questions, writing the grant, advising the team at different stages e.g. advisory panel, etc. developing research instruments, data collection, analysis or synthesis, dissemination)?
   - on-off or continued?

5. Have your co-design approaches were underpinned by existing frameworks or theories? If yes, can you tell me more about it? Which framework? Reasons for using it?

   [showing the two printed frameworks to have a look to comment and add activities]

6. What factors have facilitated research co-design in your project? can you please provide examples?

[Probe: project team and knowledge about co-design approaches, training for researchers or communities to uplift co-design skills, building trust and relationships with communities]

How successful do you think these approaches have been?
Are there things that you would have done differently?

7. What factors do you see as barriers to research co-design?

[Probe: difficulties in engaging with project target populations, lack of knowledge and interest in co-design, power imbalance, conflict of interests, time-consuming)

How did you deal with these issues?

Are there thinks that you would have done differently to minimise these barriers?

8. Have you been able to evaluate activities to see whether co-design activities achieved their promises?

   Can you comment on the project and team's capacity to capture the impact and value for end-users in this project?

9. How do you think CFI could provide support to enhance research co-design in the future?

10. For those new to research co-design, are there any resources (papers, people, websites, etc) that you would like to suggest?

11. If you want to give one tip of advice in relation to research co-design, that would that be?
